# Supplementary material for: The interactome of intact mitochondria by cross-linking mass spectrometry provides evidence for coexisting respiratory supercomplexes
Source: Mol Cell Proteomics. 2017 Dec 8;17(2):216–32. doi: 10.1074/mcp.RA117.000470 (PMC5795388; doi:10.1074/mcp.RA117.000470)
Supplement: Supplemental Data [file supp_RA117.000470_133922_0_supp_23978_fzffwf.zip › spectra_annotation/mito_spectra_annotation/490-1-4-1-2-1.pdf]

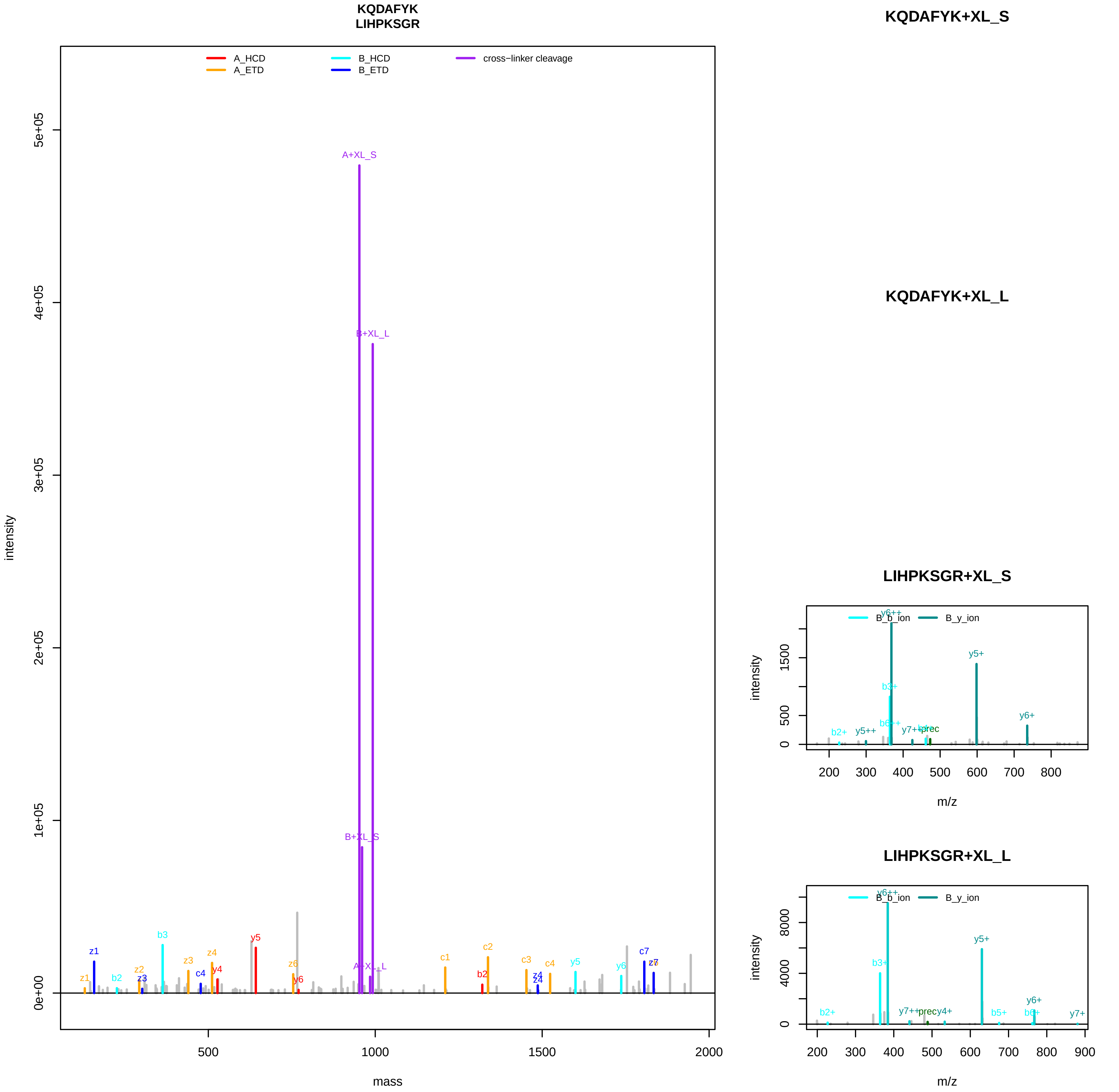
KQDAFYK
LIHPKSGR
A_HCD
A_ETD
B_HCD
B_ETD
cross−linker cleavage
A+XL_S
B+XL_L
B+XL_S
A+XL_L
intensity
mass
0e+00
1e+05
2e+05
3e+05
4e+05
5e+05
500
1000
1500
2000
KQDAFYK+XL_S
KQDAFYK+XL_L
LIHPKSGR+XL_S
LIHPKSGR+XL_L
B_b_ion
B_y_ion
m/z
y6++
y5+
b3+
y6+
b2+
y5++
y7++
prec
y4+
b5+
y7+
0
500
1500
4000
8000
200
300
400
500
600
700
800
900
